# Two Receptors, Two Isoforms, Two Cancers: Comprehensive Analysis of KIT and TrkA Expression in Neuroblastoma and Acute Myeloid Leukemia

**DOI:** 10.3389/fonc.2019.01046

**Published:** 2019-10-18

**Authors:** Timofey D. Lebedev, Elmira R. Vagapova, Vladimir I. Popenko, Olga G. Leonova, Pavel V. Spirin, Vladimir S. Prassolov

**Affiliations:** Department of Cancer Cell Biology, Engelhardt Institute of Molecular Biology, RAS, Moscow, Russia

**Keywords:** KIT, TrkA (tropomyosin receptor kinase), neuroblastoma, acute myeloid leukemia, alternative splicing, SCF (stem cell factor), NGF (nerve growth factor), oncogenic signature

## Abstract

Pediatric cancers represent a wide variety of different tumors, though they have unique features that distinguish them from adult cancers. Receptor tyrosine kinases KIT and TrkA functions in AML and NB, respectively, are well-characterized. Though expression of these receptors is found in both tumors, little is known about KIT function in NB and TrkA in AML. By combining gene enrichment analysis with multidimensional scaling we showed that pediatric AMLs with t(8;21) or inv16 and high *KIT* expression levels stand out from other AML subtypes as they share prominent transcriptomic features exclusively with KIT-overexpressing NBs. We showed that AML cell lines had a predominant expression of an alternative TrkAIII isoform, which reportedly has oncogenic features, while NB cell lines had dominating TrkAI-II isoforms. NB cells, on the other hand, had an abnormal ratio of KIT isoforms as opposed to AML cells. Both SCF and NGF exerted protective action against doxorubicin and cytarabine for t(8;21) AML and NB cells. We identified several gene sets both unique and common for pediatric AML and NB, and this expression is associated with KIT or TrkA levels. *NMU, DUSP4, RET, SUSD5, NOS1*, and *GABRA5* genes are differentially expressed in NBs with high KIT expression and are associated with poor survival in NB. We identified *HOXA10, BAG3*, and *MARCKS* genes that are connected with TrkA expression and are marker genes of poor outcome in AML. We also report that *SLC18A2, PLXNC1*, and *MRPL33* gene expression is associated with TrkA or KIT expression levels in both AML and NB, and these genes have a prognostic value for both cancers. Thus, we have provided a comprehensive characterization of TrkA and KIT expression along with the oncogenic signatures of these genes across two pediatric tumors.

## Introduction

Leukemia and neuroblastoma (NB) are the most common extracranial childhood cancers, constituting around 25 and 7% of all pediatric cancers, respectively (1, 2). NBs are responsible for 15% of cancer-related pediatric deaths and high-grade NB patients have a long-term survival rate of <40%. Although AML is generally considered a geriatric disease, it makes up 15–20% of all acute pediatric leukemias (2). Compared to the most common pediatric leukemia—acute lymphoblastic leukemia (ALL)—patients with AML have lower survival rates. Receptor tyrosine kinases and their respective ligands are one of the major drivers of tumor cells proliferation, survival, and possible differentiation. Here we explored the role of two such kinases—receptor tyrosine kinase KIT and tropomyosin receptor kinase A (TrkA)—for both leukemic and NB cells.

Receptor tyrosine kinase KIT, which binds the stem cell factor (SCF), is mainly considered as a myeloid cell receptor since it is predominantly expressed by myeloid progenitor cells and is essential for normal hematopoiesis (3). High KIT expression is a hallmark of AML and most AML cells are KIT-positive (4). CBF-AML patients have elevated KIT expression and a higher mutation rate in the gene compared to other subtypes of AML. Mutations in the KIT gene are associated with poor prognoses in patients with t(8;21)(q22;q22) and inv(16)(p13;q22) CBF-AML (5). The clinical significance of KIT overexpression is still unclear. In AML cells several KIT isoforms, which are the result of alternative splicing, are detected. Two isoforms differ by tetrapeptide sequence–GNNK presence in the juxtamembrane region of the extracellular domain (6, 7).

KIT is expressed in a significant amount of NB tumors and cell lines (8–14). According to histological studies, up to 30% of all NBs are KIT-positive, and KIT mRNA is detected in up to 80% of tumors. About half of KIT-positive NBs also co-express SCF (9, 11). Normally, in embryos, KIT is expressed by neural crest cell, from which NB originates. In neural crest cells SCF/KIT signaling drives their migration, survival, and proliferation and KIT expression is generally lost by these cells during their differentiation (15–21). Recent meta-analysis showed that high KIT expression in NBs correlates with worse prognoses independently from a tumor stage (8, 9, 11, 13, 22). KIT-positive NB cells give rise to more aggressive tumors, and KIT is currently considered as a potential therapeutic target for NB treatment. There are several FDA-approved KIT inhibitors, such as imatinib and dasatinib (8, 9, 13, 23, 24).

TrkA is a high-affinity receptor of neural growth factor (NGF) and its expression patterns and function are best characterized for cells and tissues of neural origin. TrkA is usually expressed in low-risk NBs prone to spontaneous regression (25–27). High TrkA expression and lack of *MYCN* gene amplification are associated with a favorable prognosis, whereas TrkA expression is either absent or strongly reduced in aggressive NB (28, 29). Although the expression of TrkA is generally a favorable factor, the alternatively spliced TrkAIII isoform is expressed predominantly in aggressive NBs (30). This isoform is formed as a result of alternative splicing and lacks exons 6, 7, and 9, which leads to the loss of one of two extracellular immunoglobulin-like domains and a glycosylation site. As a result of the deletion of one of the immunoglobulin-like domains, the TrkAIII isoform is constitutively active and does not respond to NGF. TrkAIII is considered to be potentially oncogenic because NB cells with TrkAIII overexpression give rise to more aggressive tumors in mice, and TrkAIII promotes angiogenesis in tumors, reduces the sensitivity of NB cells to doxorubicin, and helps cells adapt to stress (30, 31). However, this isoform is expressed not only by NB cells, but also by neural stem cells and nerve crest progenitor cells.

Expression of Trk-receptor family members was observed in several non-neural cell types and tissues. Elevated expression of TrkA is associated with a more favorable outcome and longer overall survival among breast cancer patients (32). Cutaneous melanoma cells overexpress TrkA and this is associated with poor outcomes and shorter survival (33, 34). TrkA expression is observed in lymphoid and hematopoietic cells, and its signaling is essential for immune cells (35, 36). Ectopic expression of the RUNX1-RUNX1T fusion gene, formed as a result of t(8;21) translocation common in pediatric AML, in CD34+ hematopoietic cells induces TrkA expression (37). Recently it was shown that an oncogenic TrkAIII splice isoform was expressed in the thymus and cutaneous melanomas, as well as in the Jurkat T-ALL cell line (38, 39).

In this study, we aimed to identify *KIT* and *NTRK1* (which encodes TrkA protein) gene expression patterns in pediatric patients with NB and AML (from publicly available datasets) and reveal the hallmarks of the high and low expressions of those genes. We hypothesized that in some cases the examination of the expression level of KIT and TrkA receptors is insufficient for understanding leukemia and NB cell behavior in the presence of exogenous proteins, NGF, and SCF. We characterized KIT and TrkA alternatively spliced isoform expression in NB and AML cells, as well as gene expression signatures associated with their expression, both unique and mutual for NBs and AMLs, to uncover new aspects of their signaling in pediatric tumors.

## Results

### NB and AML Have Distinct Pattern of KIT and NTRK1 Genes Expression

**Figure 1:**
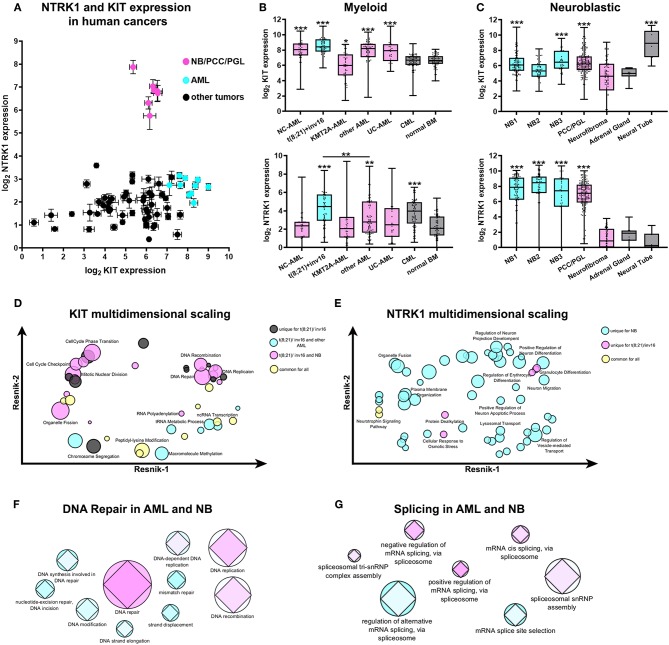
Expression and transcriptomic signature of *KIT* and *NTRK1* genes in AML and NB. **(A)** Expression of *KIT* and *NTRK1* expression across different data sets of human cancers of neurological and hematopoietic origin from R2: Genomics analysis and visualization platform (http://r2.amc.nl). Data presented as mean expression within each data set and standard errors. **(B)** Expression of *KIT* (top) and *NTRK1* (bottom) genes in pediatric AML, CML, and normal bone marrow (BM). Data on pediatric AML was taken from the denBoer data set. NC- normal cytogenetics (*n* = 39); t(8;21)/inv16- AML with translocation t(8;21) or inversion inv16 (*n* = 55); KMT2A -AML- AML with KMT2A rearrangement (*n* = 47); other AML- AML with other karyotypes (*n* = 71); UC- unknown cytogenetics (*n* = 25); CML- chronic myeloid leukemia (*n* = 69). Data on CML and normal BM (*n* = 71) were obtained from the leukemia Mile-2004 data set from the R2 platform. **(C)** Expression of *KIT* (top) and *NTRK1* (bottom) genes in NB data sets, pheochromocytomas, and paragangliomas (PCC/PGL), neurofibroma, adrenal gland, and neural tube. Data sets used for analysis: NB1- Versteeg NB data set (*n* = 88); NB2- Hiyama NB data set (*n* = 51); NB3- DeLattre NB data set (*n* = 34); PCC/PGL- Favier data set (*n* = 188), neurofibroma- Miller data set (*n* = 88), adrenal gland- from GSE3526, GSE7307, GSE8514 data sets (*n* = 13), neural tube- Nagy data set (*n* = 9). All AML and CML datasets were compared to normal BM, and NB datasets were compared to the adrenal gland. ^*^p < 0.05, ^**^p < 0.01, ^***^p < 0.001 as calculated by Mann-Whitney test. **(D)** Multidimensional scaling of enriched GO gene sets identified in t(8;21)/inv16 AML with high *KIT* expression. **(E)** Multidimensional scaling of enriched GO gene sets identified in t(8;21)/inv16 AML and NB with high *NTRK1* expression Semantic distance was analyzed by REVIGO using Resnik scores for depth of GO gene annotations. **(F)** Graphical representation of involvement of DNA damage repair or splicing **(G)** pathways in the progression of AML and NB. Colors from cyan to pink represent the prognostic value of each gene set/ Prognostic value was calculated as a normalized percentage of genes in a set correlating with prognosis. The AML data set is presented by circles and NB by diamonds. Size is proportional to the number of genes in each GO gene sets.

We examined *KIT* and *NTRK1* gene expression using the publicly available R2: Genomics analysis and visualization platform (http://r2.amc.nl) in patients with cancers of neurological [NB, glioma, pheochromocytoma (PCC), and paraganglioma (PGL)] or hematological origins (AML, ALL, CLL, lymphoma, and myeloma). NBs, along with PCC/PGL and AML datasets, form distinct groups, as shown on the plot of *KIT* and *NTRK1* gene expression (Figure 1A). Expressions of *KIT* and *NTRK1* genes for each used data set are provided in Supplementary Table 1. Next, we compared *KIT* and *NTRK1* expression for NB and AML subtypes with other tumors of similar origin and normal tissue. For NB we used the Versteeg data set (40), and for pediatric AML we used the denBoer-237 data set (41) as transcriptomic data in these two data sets was obtained and normalized by the same methods. In acute myeloid tumors, *KIT* is overexpressed in most pediatric AML karyotypes (excluding AML with KMT2A-rearrangement) compared to CML or normal bone marrow (Figure 1B). Pediatric AMLs have the same level of *NTRK1* expression as normal bone marrow, expect for AMLs with t(8;21) translocation or chromosomal inversion inv16 (Figure 1B). *KIT* expression in NB or PCC/PGL (where all three tumors originate from neural crest cells) seems to be higher than in benign neurofibromas or a normal adrenal gland (the most common site of NB localization), but lower than in the embryonic neural tube (Figure 1C). Interestingly, even though high *NTRK1* expression is strongly associated with a favorable prognosis, NB and PCC/PGL have dramatically higher *NTRK1* expression than neurofibroma, adrenal gland, or neural tube (Figure 1C).

To compare possible cellular processes associated with KIT and TrkA in AMLs and NB we used the GSEA approach to find enriched Gene Ontology (GO) gene sets in patients with high *KIT* or *NTRK1* expression (all enriched gene sets are presented in Supplementary Tables 2, 3). Since AMLs with t(8;21)/inv16 have significantly higher *NTRK1* expression than other AML karyotypes and are associated with *KIT* overexpression we decided to analyze this group separately from other AML karyotypes (including AMLs with unknown and normal cytogenetic). GSEA analysis revealed that 44 GO gene sets were enriched in t(8;21)/inv16 AMLs with high KIT expression. Unexpectedly, a substantial fraction of these gene sets (17/44) was also enriched in NBs with high KIT expression, and only 7/44 were enriched in other AML karyotypes, while another 10 gene sets were enriched in all three groups. To better elucidate biological processes shared by t(8;21)/inv16 AML and NB we performed multidimensional scaling using REVIGO (42). Multidimensional scaling of GO gene sets enriched in t(8;21)/inv16 AMLs with high KIT expression showed that KIT expression in this AML subgroup as well as in NB is associated with increased activity in DNA damage repair and cell cycle regulation pathways (Figure 1D). Surprisingly, these pathways were not enriched in other AML karyotypes, suggesting that KIT has more similar functions in t(8;21)/inv16 AML and NB, rather than in t(8;21)/inv16 and other AML karyotypes.

Next, we compared enriched GO gene sets for high *NTRK1* expression in NB, t(8;21)/inv16, and other AML karyotypes data sets. Fifty six gene sets were enriched in the NB data set, 7 in t(8;21)/inv16, and no enrichment was observed for other AML karyotypes (Figure 1E). High *NTRK1* expression in NBs was associated mainly with normal neural functions such as dendrite and neurite extension and the control of protein transport and localization in different cell compartments. In t(8;21)/inv16 AMLs, high *NTRK1* expression was mainly associated with erythrocyte and granulocyte differentiation.

To investigate if DNA damage repair processes shared for t(8;21)/inv16 AML and NB (as was revealed by multidimensional scaling) have any impact on disease outcome we analyzed how many genes from each GO gene set were associated with patient survival. We used the Kaplan Meier scan from the R2: Genomics analysis and visualization platform to find genes associated with a prognosis in two data sets: Bohlander (*n* = 422) (43) for AML and Versteeg (*n* = 88) (40) for NB. The DNA repair GO gene set was enriched in genes associated with prognosis both for AML and NB (Figure 1F). Since KIT and TrkA both have well-described alternatively spliced isoforms we also examined the relevance of different splicing pathways to prognosis for NB and AML. We chose seven GO gene sets associated with splicing and found that mRNA cis-splicing via spliceosome had the biggest amount of genes; these expressions were connected with prognoses in NB and AML (Figure 1G). These results indicate that certain DNA damage repair and splicing pathways may be relevant to prognoses in the case of both AML and NB.

### KIT Isoforms Have Different Expression Ratios in AML and NB Cells

**Figure 2:**
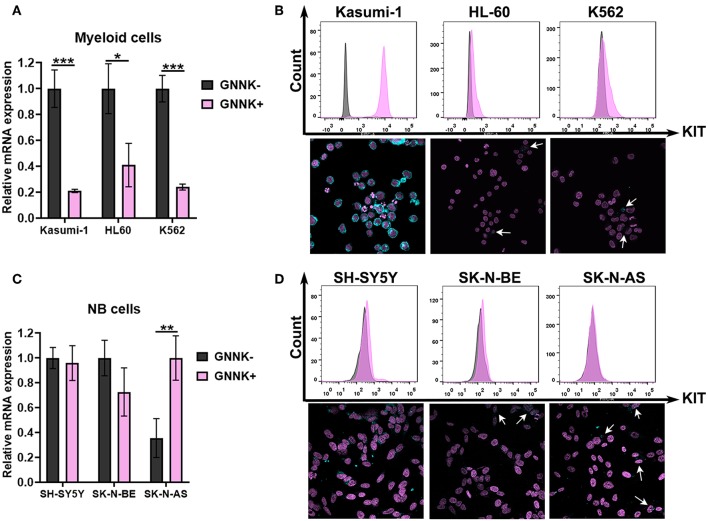
KIT expression in AML and NB cells. **(A)** Relative mRNA expression of *KIT* splice isoforms (GNNK- and GNNK+) in myeloid leukemic cell lines Kasumi-1, HL-60, and K562. **(B)** TrkA protein expression by direct flow cytometry and sub-cellular distribution of TrkA in leukemia cell lines using ICC. **(C)** Relative mRNA expression of *KIT* splice isoforms (GNNK- and GNNK+) in NB cell lines SH-SY5Y, SK-N-BE, and SK-N-AS. **(D)** TrkA protein expression by direct flow cytometry and sub-cellular distribution of TrkA in NB cell lines using ICC. ^*^p < 0.05, ^**^p < 0.01, ^***^p < 0.001 as calculated by the Mann-Whitney test.

First, we measured the mRNA expression of two KIT isoforms (GNNK+ and GNNK-) in three myeloid leukemia (Kasumi-1, K562, and HL-60) and three NB (SH-SY5Y, SK-N-BE, and SK-N-AS) cell lines. All tested AML and NB cell lines had detectable mRNA expression of both KIT isoforms. GNNK- was the predominant isoform in all leukemic cell lines, but in the HL-60 cell line the differences between GNNK- and GNNK+ transcripts were the smallest (Figure 2A). We then stained three myeloid leukemia cell lines with an anti-KIT (conjugated with FITC) antibody to determine the expression of KIT on protein level. Direct flow cytometry analysis of alive leukemia cells confirmed that 100% of Kasumi-1 cells had surface KIT expression (44), and K562 cells had a sizeable KIT-positive population. In agreement with previous research, HL-60 had a very low level of surface KIT (45), and only intracellular KIT protein was detected in this cell line after fixation and permeabilization prior staining (Figure 2B). ICC confirmed that Kasumi-1 cells have the highest KIT expression level as cytoplasm was stained intensively. Only part of the K562 and HL-60 population of K562 and HL-60 cells had considerable KIT staining.

Notably, we found that two NB cell lines, SH-SY5Y and SK-N-BE, had roughly the same ratio of GNNK- and GNNK+ KIT isoform expression, while SK-N-AS have a predominant GNNK+ isoform expression (Figure 2C). Direct flow cytometry and ICC confirmed that all NB cells expressed the KIT receptor but at different levels (Figure 2D). The highest staining intensity with anti-KIT antibodies was detected in SH-SY5Y cells, while SK-N-BE and SK-N-AS apparently had a very small population of cells with noticeable staining.

### TrkAIII Isoform Is Highly Expressed in AML Cells

**Figure 3:**
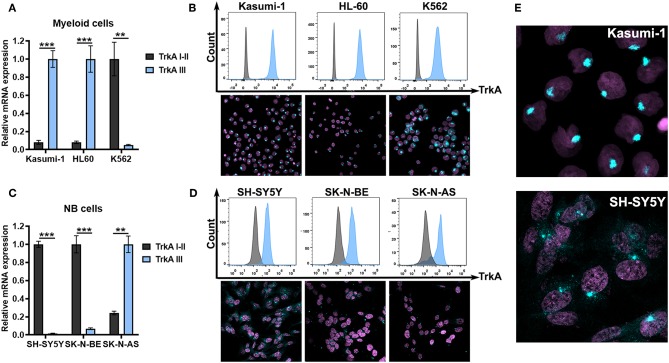
TrkA expression in AML and NB cells. **(A)** Relative mRNA expression of TrkA splice isoforms (TrkAI-II and TrkAIII) in myeloid leukemia cell lines Kasumi-1, HL-60, and K562. **(B)** TrkA protein expression by direct flow cytometry and sub-cellular distribution of TrkA in leukemic cell lines using ICC. **(C)** Relative mRNA expression of TrkA splice isoforms (TrkAI-II and TrkAIII) in NB cell lines SH-SY5Y, SK-N-BE, and SK-N-AS. **(D)** TrkA protein expression by direct flow cytometry and sub-cellular distribution of TrkA in NB cell lines using ICC. **(E)** Larger images of TrkA sub-cellular distribution of Kasumi-1 and SH-SY5Y cells. ^**^p < 0.01, ^***^p < 0.001 as calculated by the Mann-Whitney test.

Further, we examined NTRK1 isoform expression in myeloid and neuroblastoma cell lines. Kasumi-1 and HL-60, which represent the M2 subtype of AML, highly express TrkAIII isoforms (Figure 3A). We then stained three myeloid leukemia cell lines with anti-TrkA antibodies. For flow cytometry and ICC examination of TrkA protein expression, we used FITC-conjugated antibodies that targeted the tyrosine kinase domain of the receptor, shared by all three isoforms. Cells were pre-fixed and permeabilized, and in all leukemic cells we detected a significant amount of intracellular receptors. We used an ICC approach to determine TrkA distribution within the cells (Figure 3B). The pattern of TrkA distribution in Kasumi-1 and HL-60 cells differed dramatically from that observed in K562 cells. In K562 cells a lot of protein localized all over the cellular cytoplasm, while in Kasumi-1 and HL-60 cells TrkA protein localized to circular-shaped, spike-like compartments, near the nuclei (Figure 3E). The size of the TrkA-spikes was notably smaller in HL-60 cells than in Kasumi-1.

SH-SY5Y and SK-N-BE cells have a dramatically higher expression of TrkAI-II isoforms than TrkAIII isoforms (Figure 3C). SK-N-AS cells, on the contrary, have higher TrkAIII expression. All NB cells seem to express TrkA proteins as detected by flow cytometry. ICC showed a more complicated TrkA expression pattern in the NB cell. SH-SY5Y had the brightest staining and a significant amount of the receptor was localized to the cytoplasm or membrane (Figures 3D,E). In leukemic cells we also detected spikes that were close to the nuclei. SK-N-BE and SK-N-AS had little amounts of staining in the cytoplasm with the majority of cells having only spikes.

### SCF and NGF Promote Survival of Both AML and NB Cells

**Figure 4:**
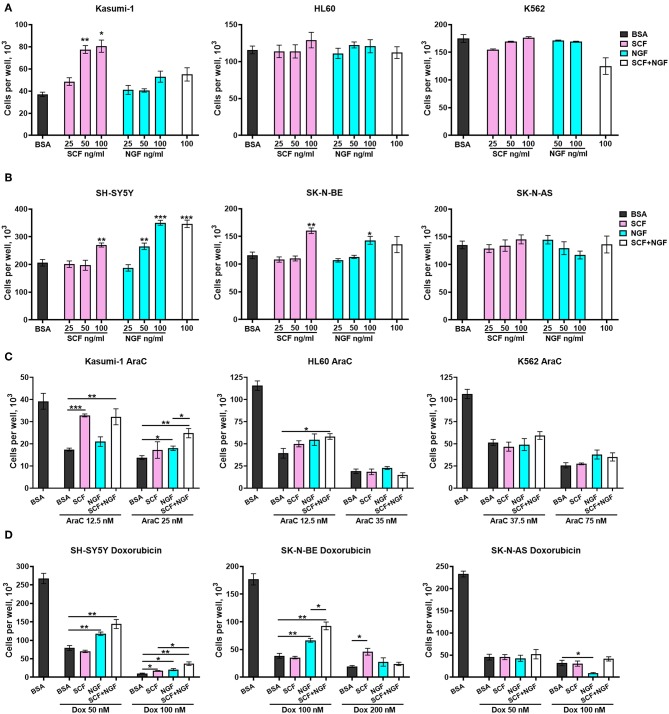
Growth and survival of AML an NB cells in the presence of NGF and SCF. Number of viable **(A)** leukemic cells and **(B)** NB cells in response to treatment with recombinant proteins—SCF (25–100 ng/ml), NGF (25–100 ng/ml) or their combination (100 + 100 ng/ml). **(C)** Number of viable leukemic cells in response to treatment with cytarabine (AraC) in the presence of recombinant proteins. **(D)** Number of viable leukemic cells in response to treatment with doxorubicin (Dox) in the presence of recombinant proteins. Leukemic and NB cells were pretreated with recombinant SCF (100 ng/ml), NGF (100 ng/ml), or their combination (100 + 100 ng/ml). Two concentrations of AraC or doxorubicin were used for each cell line. Data presented as mean number of viable cells per well with SD. ^*^p < 0.05, ^**^p < 0.01, ^***^p < 0.001 as calculated by the Mann-Whitney test.

To explore the role of TrkA and KIT isoforms in NB and AML cells, we first tested whether receptor activation by SCF or NGF affects the proliferation of cells with different ratios of receptor isoforms. NB and leukemic cell lines were treated with different concentrations of SCF or NGF (25–100 ng/ml) alone and with 100 ng/ml of both SCF and NGF, with an addition of even low concentrations (25 ng/ml) of SCF stimulated growth of Kasumi-1 cells that had the highest level of KIT expression (Figure 4A). None of the analyzed myeloid cell lines responded to NGF treatment by elevated growth rate. Consistent with previous reports, NGF did not drive K562 cell line growth despite the high expression of full-length TrkA receptor isoforms. Surprisingly, the addition of NGF to SCF-treated Kasumi-1 cells resulted in the abolishment of SCF-induced growth. As it was shown on cytokine-dependent cell line TF-1, NGF/TrkA signaling is not preferred in hematopoietic cells and even trace amounts of GM-CSF could nullify NGF effects (46).

Both NGF and SCF were able to stimulate the proliferation of NB cell lines. SCF (at 100 ng/ml) stimulated only SH-SY5Y and SK-N-BE cell proliferation (Figure 4B). NGF promoted growth of SH-SY5Y cells (at 50 and 100 ng/ml) and increased the proliferation of SK-N-BE cells only at 100 ng/ml. Both SCF and NGF failed to promote the growth of SK-N-AS cells. These results are in agreement with previous publications stating that TrKAIII isoforms (dominant in SK-N-AS) cannot interact with NGF (30). The absence of SCF stimulating effects on SK-N-AS cells may be connected to SK-N-AS having a higher expression of KIT GNNK+ isoforms over canonical GNNK-, since GNNK+ isoforms show weaker responses to SCF in other studies (6, 7). NGF and SCF showed no synergic action on the proliferation of NB cell lines.

To investigate what role SCF/KIT and NGF/TrkA signaling might play in NB and AML progression we studied how SCF and NGF protect cells against commonly used chemotherapeutic drugs (cytarabine-AraC and doxorubicin). Cells were pretreated with recombinant proteins SCF and NGF either individually (100 ng/ml) or in combination (100 ng/ml each) and then treated by AraC (for leukemic cells) or doxorubicin (for NBs) for 6 days. For each cell line, we used two toxic concentrations of the respective drug, representing mildly and highly toxic concentrations. A combination of NGF and SCF protects leukemic cells Kasumi-1 and HL-60 from AraC-induced cell death (Figure 4C). Interestingly, when Kasumi-1 cells are treated with a lower concentration of AraC (12.5 nM), SCF alone is sufficient, but only NGF in combination with SCF can stimulate the survival of those cells treated with high AraC concentrations. The effect of NGF and SCF combination is reproduced in HL-60 cells under slightly toxic AraC concentrations (12.5 nM). Unexpectedly, neither SCF nor NGF affected K562 cells treated with AraC.

In a similar manner to proliferation stimulation, NGF protected NB cells with dominant TrkAI-II isoforms (SH-SY5Y and SK-N-BE) from doxorubicin, especially from lower doxorubicin concentrations (Figure 4D). NGF promoted the death of SK-N-AS cells (doxorubicin 100 nM) that had a dominant TrkAIII isoform. While SCF failed to protect NBs from lower doxorubicin concentrations it was able to protect cells from high doxorubicin concentrations (100 nM for SH-S5Y5 and 200 nM for SK-N-BE). SCF and NGF showed synergic protective action from doxorubicin (100 nM) for SH-SY5Y and SK-N-BE cells. As is consistent with our previous results, SCF alone or together with NGF did not affect the survival of SK-N-AS cells.

These results reveal that both SCF and NGF can promote survival of AML and NB cells, but still have distinct action on the cells dependent on the dominating isoform of KIT or TrkA receptors. Importantly, SCF and NGF showed combined promotion in the survival of both AML and NB cells.

### Novel Prognostic Factors in AML and NB Patients Are Associated With KIT and NTRK1 Expression

**Figure 5:**
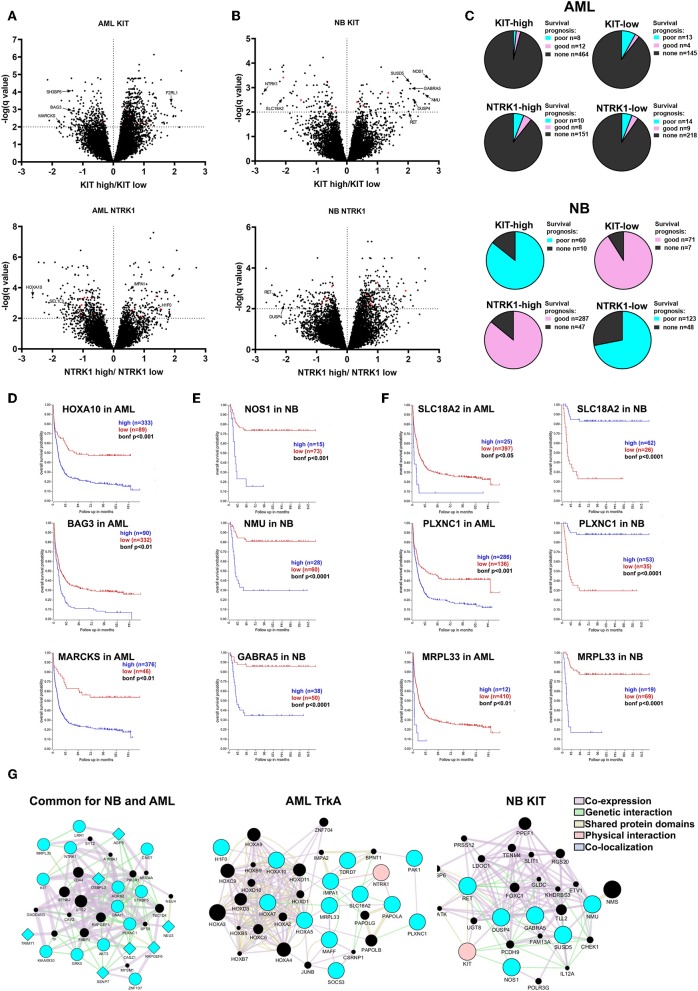
Evaluation of prognostic markers in AML and NB patients depending on *KIT* or *NTRK1* expression level. Volcano plots for differentially expressed genes (DEGs) in AML **(A)** and NB **(B)** groups with KIT high/low and NTRK1 high/low expression. DEGs common for both AML and NB are marked red. **(C)** Distribution of DEGs in AML (top) and NB (bottom) groups based on correlation with prognosis. **(D)** Kaplan Meier survival analysis for *HOXA10, BAG3*, and *MARCKS* genes expression in AML data set (Bohlander *n* = 422). **(E)** Kaplan Meier survival analysis for *NOS1, NMU*, and *GABRA5* genes expression in NB data set (Versteeg *n* = 88). **(F)** Kaplan Meier survival analysis for *SLC18A2, PLXNC1*, and *MRPL33* genes expressed in AML and NB data sets. **(G)** Gene and protein interaction networks generated for identified gene sets using GeneMANIA. Blue nodes are input genes from identified gene sets. Black nodes are result genes identified by GeneMANIA that may participate in gene/protein interactions. Genes identified as associated with *NTRK1* expression are presented as circles and genes associated with *KIT* expression as diamonds.

To determine what novel prognostic factors are associated with *KIT* or *NTRK1* expression in AML and NB we identified differentially expressed genes (DEGs) in patient groups with high *KIT* or *NTRK1* expression (Figures 5A,B). Complete lists of identified DEGs are presented in Supplementary Tables 4, 5. We then examined the correlation of identified DEGs expression with prognoses in AML (Bohlander-422 data set) and NB (Versteeg-88 data set) by Kaplan Meier scan in R2: Genomics analysis and visualization platform (Figure 5C).

In the *KIT*-high group we detected 689 DEGS, and among them 20 were associated with a prognosis in AML (Bohlander data set). Two hundred sixty-four genes were found in the *KIT-*low group and 17 were associated with a prognosis. Two hundred twenty-two DEGs were found associated with *NTRK1* high expression and 18 were linked with a prognosis. Three hundred thirty-six DEGs were in *NTRK1*-low group and 23 connected with prognosis. Among identified DEGs we found several novel prognostic genes, those expressions strongly correlate with a poor prognosis. In the *KIT*-low group we marked *BAG3, SH3BP5*, and *MARCKS* genes as their high expression is associated with a poor prognosis (Figure 5D). *BAG3* (BCL2-associated athanogene 3) knockdown is known to affect proliferation, migration, and invasion of EGFR-positive triple-negative breast cancer cell lines via AKT and FAK kinases (47). *MARCKS* (Myristoylated alanine-rich C kinase substrate) is involved in the tumorigenesis of different types of malignant diseases, but its role in myeloid malignances has not been described. *F2RL2* (coagulation factor II (thrombin) receptor-like 1) is a gene encoding G-protein coupled receptor and it is differentially expressed in the *KIT*-high group. It is involved in inflammatory processes, but its function in myeloid hematopoiesis is not described. We found three HOX genes (*HOXA5, HOXA7, HOXA10*) among DEGs in the *NTRK1*-low group. HOX genes encode transcription factors of the homeobox family, implicated in normal hematopoiesis and several malignancies including ALL and AML (48). Even so, it is known that the *HOXA10* gene has oncogenic potential in developing leukemia; specific roles of *HOXA5, HOXA7*, and *HOXA10* in myeloid leukemias are poorly described. Among genes associated with *NTRK1* expression we identified several novel genes connected with poor prognosis in AML that were not previously described: *H1F0, SEL1L3*, and *IMPA1*. Here, we, for the first time, found that *H1F0* (coding linker histone H1.0) is linked to high *NTRK1* expression. In contrast to AML, low expression of *H1F0* in glioblastoma, melanoma, and other types of cancer is a marker for poor clinical outcomes (49). A clear description of *SEL1L3* [SEL1L3 sel-1 suppressor of lin-12-like 3 (*C. elegans*)] function in cancer cells does not exist, but we observed that its high expression is also associated with a poor prognosis in AML. High expressions of *IMPA1* that encode inositol(myo)-1(or 4)-monophosphatase 1 are also markers of poor outcomes in AML. Previously, its role was described only in neural cell behavior but not in leukemic cells (50).

Seventy-six and Seventy-eight DEGs were identified in NB with *KIT-*high and low expression, respectively (Figure 5B). The majority of DEGs from *KIT*-high and *NTRK1*-low populations are associated with a poor prognosis, while genes identified in *KIT*-low and *NTRK1*-high populations are associated with a favorable prognosis (Figure 5C). Several genes, like *RET, HIF1a*, and *DUSP4* were detected both in *KIT*-high and *NTRK1*-low groups. Both receptor tyrosine kinase RET and dual-specificity kinase DUSP4 are implicated in ALK oncogenic action via ERK-ETV5-RET signaling, and their high expression is associated with a poor prognosis in NBs (51, 52). In patients with high KIT expression we also identified *NOS1, NMU*, and *GARBA5* genes, and these expressions are specific to neural tissue and are also associated with poor disease outcome (Figure 5E). *NOS1* encodes neuronal nitric oxide synthase, and it is reported that high *NOS1* expression may be responsible for PC12 pheochromocytoma cell resistance to NO-induced toxicity (53). The possible role of neuromedin U (*NMU*) or GABA type A receptor subunit alpha-5 (*GABRA5*) in NB progression was not previously described, but there are reports that *NMU* is overexpressed and involved in the progression of pancreatic, breast, and endometrial cancers (54–56). We also identified several differentially expressed genes that are associated with *KIT* or *NTRK1* expression in both NB and AML datasets. Several of these genes were associated with prognoses, most notably *MRPL33, SLC18A2*, and *PLXNC1* (Figure 5F). To determine possible connections between identified DEGs, we employed GeneMANIA analysis. We analyzed three gene sets: DEGs mutual to NB and AML, subset of genes associated with *NTRK1* expression level in AML, and DEGs associated with poor prognosis and high KIT expression in NB. This analysis revealed that genes in all three sets formed interconnected networks, suggesting that there are multiple possible interactions between identified genes.

**Figure 6:**
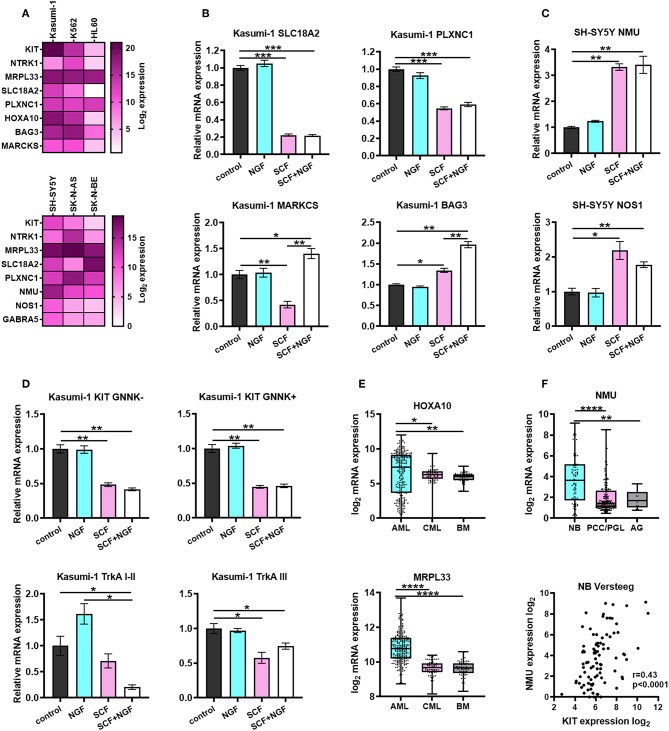
Characterization of prognostic marker genes expression in leukemic and NB cells. **(A)** Heatmap of normalized expression of *KIT, NTRK1, MRPL33, PLXNC1, SLC18A2, HOXA10, BAG3, MARCKS, NMU, NOS1*, and *GABRA5* genes in leukemic and NB cell lines. Expression of each gene was normalized to *GAPDH* expression in the respective cell line. *KIT* and *NTRK1* expression is presented as a summarized expression of all mRNA isoforms. **(B)** Expression of *PLXNC1, SLC18A2, BAG3*, and *MARCKS* in Kasumi-1 cells treated with SCF, NGF or both. **(C)** Expression of *NMU* and *NOS1* in SH-SY5Y cells treated with SCF, NGF, or both. **(D)** Expression of KIT and TrkA isoforms in Kasumi-1 cells treated with SCF, NGF, or both. **(E)** Expression of *HOXA10* and *MRPL33* genes in pediatric AML patients (denBoer dataset), CML and normal bone marrow (BM) from Mile-2004 data set. **(F)** Expression of *NMU* gene (top graph) in NB patients (Versteeg dataset), pheochromocytomas and paragangliomas (PCC/PGL), and the adrenal gland (AG). Spearmen correlation of NMU and KIT expression in NB patients (Versteeg dataset). ^*^p < 0.05, ^**^p < 0.01, ^***^p < 0.001, ^****^p < 0.0001 as calculated by the Mann-Whitney test.

To verify gene expression patterns associated with TrkA and KIT, we measured *MRPL33, SLC18A2, PLXNC1, HOXA10, BAG3, MARCKS, NMU, NOS1*, and *GABRA5* in myeloid and NB cells (Figure 6A). MRPL33 expression is a prognostic marker mutual to NB and AML and is highly expressed in all AML and NB cell lines. The total TrkA and KIT expression level is similar in K562 and Kasumi-1 cells, being rather distinct from that observed in HL-60 (Figure 6A).

To determine if the expression of identified DEGs is dependent on the activation of KIT or TrkA we treated Kasumi-1 and SH-SY5Y cells with SCF (100 ng/ml), NGF (100 ng/ml), or both (Figures 6B–D). Proteins were added to cell cultures for 72 h, followed by a second treatment for 6 h to measure both possible long-term and short-term effects of SCF and NGF on the expression of identified DEGs. We identified that *HOXA10* and *MRPL33* expression is elevated in AML patients compared to CML or normal BM, together with the highest level in Kasumi-1 among three studied cell lines (Figures 6A,E). However, its expression changes were not detected upon stimulation with recombinant proteins (data not shown). Interestingly, NGF+SCF and SCF alone had distinct effects on *MARCKS* mRNA levels, as its expression fell down after SCF treatment and was restored by the presence of NGF (Figure 6B). NGF strengthened effect caused by SCF regarding *BAG3* expression. As for *SLC18A2* and *PLXNC1* (identified DEGs common for NB and AML), their expression changed similarly upon SCF or NGF+SCF stimulation, which suggested a major contribution of KIT in their regulation (Figure 6B).

Treatment of TrkAIII-dominant Kasumi-1 cells with NGF alone made no significant changes in KIT and TrkA isoform expression along with the expression of identified DEGs (Figures 6B,D). Although, neither NGF nor SCF significantly affected TrkAI-II expression statistically, their combination significantly decreased TrkAI-II expression (Figure 6D). Conversely, only SCF was able to affect TrkAIII expression. These data suggest an existence of KIT-TrkA regulatory crosstalk in AML cells. Stimulation of Kasumi-1 cells with only SCF resulted in a decrease in both GNNK-/+ isoforms, and the combination did not exhibit any additional effects (Figure 6D).

We observed that *NMU, NOS1*, and *GABRA5* expression is highest in SH-SY5Y cells, which have the highest *KIT* expression among NB cell lines (Figure 6A). These results are in an agreement with all these genes being associated with high *KIT* expression in NB patients. More importantly, the expression of *NMU* and *NOS1* was elevated after the treatment of SH-SY5Y with SCF (Figure 6C). We did not observe any significant changes in expression of other genes after treatment of SH-SY5Y with SCF, NGF, or both (data not shown). *NMU* expression is also elevated in NB tumors compared to PCC/PGL or normal adrenal glands and correlates with *KIT* expression in NB patients (Figure 6F).

## Discussion

The role of TrkA and KIT signaling in AML and NB is poorly understood. KIT and TrkA staining of leukemic and NB cells showed similar results with the regard to the expression of alternative receptor isoforms. NB cells have either an equal ratio of KIT GNNK– and GNNK+ isoforms or a predominant expression of GNNK+, while leukemic cells express substantially more GNNK– than GNNK+ isoforms. The significance of the GNNK+/– ratio in normal and malignant cells is still not precisely described in published data. It is stated that GNNK- isoforms are predominant in normal KIT-positive tissues. We have identified that GNNK+ isoforms are expressed at the same or higher level as GNNK- in NB cells. Since KIT is associated with a poor prognosis in NB but not in AML, we speculate that the ratio of splice isoform can be responsible for the oncogenic potential of the non-mutant KIT. According to several reports, KIT-positive cells in NB tumors (as opposed to AML) have cancer stem-like properties (57–60). Considering that both AML and NB cells express GNNK+, and that the GNNK-/GNNK+ expression ratio is much lower in NB cells, we speculate that GNNK+ isoform expression may be specific to cancer stem cells in NB and even possibly in AML.

Consistent with previous reports, in AML and CML cells we saw different levels of TrkA expression, both on mRNA and at protein level. Also, we demonstrate that TrkA isoform ratios are different between AML and CML cells. It was shown that the K562 (as well as TF-1) cell line has detectable surface TrkA expression as well as its mRNA transcript, while AML cells KG1, MOLM13, and MV4-11 expressed TrkA only at the mRNA level (46). In all studied AML cell lines, we detected intracellular TrkA (by flow cytometry) and observed that cells with a predominance of TrkAIII showed distinct distribution of TrkA within the cells. Interestingly, the TrkA in AML and NB cells was mostly localized in the vesicle compartment near the nucleus. Only SH-SY5Y and K562 cells, which have the highest ratio of TrkAI-II/TrkAIII expression among NB and leukemic cells, respectively, showed notable cytoplasm localization of the TrkA protein. The TrkAIII isoform is localized mostly to ER/Golgi compartments, and this isoform is considered oncogenic. When localized to ER/Golgi it can activate different pro-survival signaling (61, 62).

One of the hallmarks of pediatric tumors is a high prevalence of chromosomal aberrations and low incidence of point mutations. Still, the abnormal expression of different RTKs, such as TrkA and KIT, can have a substantial impact on tumor formation and progression. Unexpectedly, we found that t(8;21)/inv16 bearing AML with a high KIT expression phenotype shares more features with high KIT-expressing NB tumors than with other AML karyotypes. In both t(8;21)/inv16 AML and NB, tumors high KIT expression is strongly associated with DNA repair mechanisms and processes of cell cycle regulation. In NB tumors, high activity of DNA damage repair is associated with a worse prognosis for overall patient survival, and there are some reports that argue that targeting DNA damage repair may be beneficial for a subset of AMLs (63, 64). It is possible that high activity of DNA damage repair and NGF signaling pathways can be a transcriptomic signature of *MYCN*-amplified NB (65, 66). Moreover, NGF and SCF had similar effects on the survival of NB cells and Kasumi-1 cells with t(8;21) translocation. In both cases, NGF and SCF showed a combined protective effect against doxorubicin or cytarabine.

NGF/TrkA signaling is implicated in normal hematopoiesis, and bone marrow stromal cells produce NGF (46, 67, 68). Differentiation by retinoic acid induces the expression of TrkA receptors in myeloid cell lines (69, 70). NGF can influence the differentiation of hematopoietic cells - basophils, acting synergistically with the granulocyte-macrophage colony-stimulatory factor (GM-CSF) (71). Moreover, NGF promotes megakaryocytic differentiation but blocks erythroid differentiation (72). The role of NGF/TrkA signaling in the development or progression of myeloid malignancies is unclear. It was shown that TrkA signaling in CML cells is activated in response to a blockade of BCR-ABL1 by imatinib (73). RUNX1-RUNX1T1 knockdown in the Kasumi-1 cells leads to the upregulation of cytokine-pathways including NGF (44). The co-expression of TrkA and its ligand NGF in murine hematopoietic progenitor cells induces leukemia (74). Notably, TrkA is a common off-target for FLT3 and JAK2 inhibitors used in myeloid leukemia treatment, so leukemia patients may benefit from non-intentional TrkA inhibition along with FLT3 or JAK2 (75). Since TrkA inhibitors also block FLT3 and JAK2, the simultaneous blocking of these receptors may be considered as a prospective therapeutic approach.

Although high TrkA expression in NB is associated with a favorable prognosis, the expression of NGF may play a different role in NBs with low or moderate TrkA expression (76). In general, NBs with a favorable prognosis and high level of TrkA expression poorly express NGF. In cells obtained from patients with late-stage NBs, TrkA expression is significantly reduced, and the addition of NGF does not lead to differentiation of these cells. High NGF expression in NB is associated with a poor prognosis and it is reported that NGF stimulates NB survival under conditions of nutrient deprivation (77, 78). The protective effect of SCF on NB cells against chemotherapy drugs was not previously described, although there is much evidence to suggest that KIT inhibition leads to increased apoptosis and NB cell death. These findings highlight a possible important role of SCF and NGF co-expression in acquired drug resistance in NB and AML.

We described several gene sets, and those expressions may represent a molecular signature of KIT and TrkA expression levels in NB and AML. *PLXNC1, MRPL33*, and *SLC18A2* genes are strongly implicated in gene interaction networks common for NB and AML, and they are also associated with *NTRK1* expression levels in AML (Figure 5G). The *MRPL33* gene that encodes large mitoribosomal sub-units regulates growth and apoptosis in cancer and may also be implicated in chemoresistance (79). Notably, *MRPL33* expression correlates with a poor prognosis both in NBs and AML. The *PLXCN1* gene encoding Plexin C1-type I transmembrane receptor is a target of the Runx1 transcriptional factor that is involved in chromosomal rearrangements in AML (80). *PLXCN1* expression is associated with low *NTRK1* expression in AML and high *NTRK1* expression in NB. Also, Plexin C1 is involved in melanoma progression, its expression is absent in the early stages of the melanoma but occurs in late stages as a pro-survival and anti-apoptotic factor marker and acts via the activation of AKT pathway (81). Interestingly TrkA is considered to be a potential oncogene in malignant melanoma (33), and predominant TrkAIII expression has been detected in metastatic melanoma (39). That may point to the existence of the connection between *NTRK1* and *PLXCN1* expression in malignant diseases. SLC18A2 protein is involved in vesicular transportation of monoamines; mRNA was found in basophilic and mast cells (82). HOX proteins are involved in the regulation of myeloid cell development during embryogenesis and adult hematopoiesis. *HOXA5* expression is crucial for erythroid and myeloid commitment (83). Overexpression of the *HOXC6* gene induces gene expression programs similar to acute myeloid leukemia. DEGs for *HOXC6*-overexpressing cells include *SLC18A2* (84). We found that the high expression of *PLXNC1* and *SLC18A2* genes is associated with poor prognosis in AML and favorable prognosis in NB, and also their expression is elevated in Kasumi-1 cell lines. Obtained results give evidence that the expression of these genes is responsive to SCF treatment, suggesting their dependence on KIT-signaling. Hence, Kasumi-1 cells could be used to study *PLXNC1* and *SLC18A2* oncogenic potential in AML. Moreover, our study revealed the responsiveness of AML cells to NGF treatment as a combination of NGF and SCF was not only able to protect TrkAIII-high cells from cytarabine-induced cell death but also resulted in down-regulation of the main TrkA isoform. The treatment of Kasumi-1 cells with SCF had a distinct effect on KIT-correlated genes *BAG3* and *MARCKS; MARCKS* was down-regulated while *BAG*3 was up-regulated. NGF addition enhanced SCF-induced effects regarding *BAG3* while *MARCKS* expression was restored by it. Overall, these data slightly clarify possible mechanisms of the contribution of TrkA signaling to AML cell survival.

For NB we identified a gene/protein interaction network of six genes associated with a poor prognosis and high KIT expression in NB (Figure 5G). Although the functions of *NOS1, GABRA5*, and *NMU* genes in NB are unknown, there is some evidence of how they might affect NB progression. Neuromedin U (*NMU*) is implicated in the development of ALK inhibitor resistance in non-small cell lung cancer (85). Since RET and DUSP4 are also involved in the development of ALK inhibitor resistance and share a gene interaction network with Neuromedin U, it may play a similar role in NB development. Interestingly, Neuromedin U, along with its receptor NMU1R, is expressed by AML cells and probably stimulates the growth of primary AML cells through autocrine loop (86). Nitric oxide (NO) is reported to be an important negative regulator of *MYCN* expression during NB and neuronal differentiation (87). Loss of sensitivity to NO due to constant *NOS1* overexpression can be one of the mechanisms that block NB differentiation (53). *NOS1* expression can be increased by the stimulation of NB cells with GABA-containing liposomes, although the particular role of GABA type A receptor subunit alpha 5 (*GABRA5*) in this process is unknown (88). There are reports that GABA-A receptor agonists induce apoptosis in NB cells, although the high expression of some GABA receptor subunits correlates a with poor prognosis in NB (89, 90). We showed that *NMU* and *NOS1* expression can be upregulated by KIT activation by SCF. *NMU* expression in NB is significantly higher than in pheochromocytomas, paragangliomas, or normal adrenal glands; it correlates with KIT expression and is associated with a poor outcome, which suggests that it is a novel prognostic factor associated with KIT expression in NB.

Our findings should be valued within at least two technical aspects. First, we could not investigate the prognostic value of KIT and TrkA isoforms. The reason for this there is that no AML or NB data sets have transcriptomic or proteomic data on alternatively spliced KIT and TrkA expression as well as survival data. Secondly, we do not show whether identified DEGs expression is dependent on the expression of specific receptor isoforms. Although, this is a limitation, we first studied how DEGs expression changed in response to stimulation with SCF or NGF. In conclusion, this study determined the pattern of KIT and TrkA isoform expression in NB and AML cell lines. Nonetheless, we identified several novel transcriptomic signatures for AML and NB patients connected with KIT and TrkA expression. Our data elucidated new prognostic markers that may be involved in the progression of pediatric tumors.

## Materials and Methods

### Cell Cultures

Human AML cells HL-60, CML cells K562 (harbors BCR/ABL translocation), NB cells SH-SY5Y, SN-K-AS (without MYCN amplification), and SK-N-BE (MYCN amplified) were cultured at 37°C and 5% CO_2_ in a RPMI-1640 growth medium supplemented with 10% fetal bovine serum. Kasumi-1 cells [harbors t(8;21) translocation] were cultured in RPMI-1640 growth medium supplemented with 20% fetal bovine serum. All growth media were also supplemented with 2 mM L-glutamine, 100 units/ml penicillin, 100 μg/ml streptomycin, and 1 mM sodium pyruvate. All cell lines were gifted by the Heinrich-Pette Institute at the Leibniz Institute for Experimental Virology. RPMI-1640, penicillin/ streptomycin, sodium pyruvate, and L-glutamine were purchased from Gibco (ThermoFisher Scientific, USA).

### Direct Flow Cytometry

2^*^10^5^ cells were stained in round-bottom tubes. Cells were washed in (1% sodium azide, 1% FBS in PBS) and resuspended in 20 ul of staining buffer (3% BSA in PBS). For intracellular staining, cells were fixed in 100 ul 4% PFA for 10 min and washed with 400 ul of PBS prior to staining. Cells were permeabilized by the addition of 1% Triton X-100 to the staining buffer. Conjugated antibodies, anti-KIT antibodies conjugated with FITC (ab119107, Abcam, USA) and anti-TrkA with Alexa Fluor 488 antibody (ab194321, Abcam, USA), were diluted in staining buffer. Amounts of antibodies were added in accordance with the manufacture's protocol: 1:20 dilution in staining buffer. Cells were incubated for 30 min in the dark at room temperature and washed with ice-cold PBS once. Measurements were performed on a LSRFortessa flow cytometer (BD Biosciences) and analyzed with FlowJo software.

### Quantitative Real-Time PCR

RNA extraction from myeloid and NB cells was performed using TRIzol reagent (Invitrogen, ThermoFisher Scientific, USA) in accordance with manufacturer's protocol. The yield and purity of RNA was determined by a spectrophotometer NanoDrop ND-1000 (Thermo Scientific, USA). An amount of 2 μg of RNA were used for the synthesis of cDNA by RevertAid Reverse Transcriptase kit (Thermo Scientific, USA). Real-time PCR was performed in three replicates using the Maxima SYBR Green Supermix (Thermo Scientific, USA) and CFX96 Real-Time System (Bio-Rad, USA). The expression levels of studied genes were normalized to that of the human GAPDH. Ct values and relative expression was determined by CFX Manager 3.1 software (Bio-Rad, USA). Primer sequences used in this study are provided in Supplementary Table 6.

### Analysis of Cell Growth

Kasumi-1 (5^*^10^3^ per well), HL-60 (3^*^10^3^ per well), K562 (5^*^10^3^ per well), SH-SY5Y (15^*^10^3^ per well), SK-N-AS (10^*^10^3^ per well), and SK-N-BE (2^*^10^3^ per well) cells were seeded in triplicates in 48 well plate prior drug or recombinant proteins treatment. Six days after treatment, cells were counted on the Neubauer chamber by the trypan blue exclusion method. Doxorubicin hydrochloride (D1515) and cytosine β-D-arabinofuranoside (AraC, C1768) were purchased from Sigma-Aldrich (USA). Recombinant SCF (ab9717) and NGF (ab179616) were purchased from Abcam (USA). SCF and NGF were reconstituted in 0.1% BSA, and 0.1% BSA was used as a negative control in experiments with recombinant protein treatments. Each experiment was performed several times.

### Immunocytochemistry (ICC) and Confocal Imaging

The cells were fixed with 4% PFA in a 0.1 M phosphate buffer (pH 7.3) for 15 min, washed with PBS (3 × 10 min), treated with 0.2% Triton X-100 in PBS (10 min at room temperature), washed with PBS (10 min), and incubated overnight at 4°C with anti-KIT antibody conjugated with FITC (ab119107, Abcam, USA) or with anti-TrkA-Alexa Fluor 488 antibody (ab194321, Abcam, USA) diluted 1:50 in PBS containing 1% BSA. After three washes with PBS, the cells were mounted in Slowfade gold medium (Invitrogen, USA, cat. s36936) containing 1 μg/ml DAPI (Sigma-Aldrich, USA) and sealed with nail polish. Confocal 8-bit digital images were obtained using a Leica TCS SP5 laser-scanning microscope (Leica, Germany) equipped with an HCX PLAPO CS 63 × 1.4 oil immersion lens. The image acquisition parameters were as follows: DAPI fluorescence (DNA staining) with excitation at 405 nm and emission at 420–480 nm; FITC and Alexa Fluor 488 fluorescence, excitation at 488 nm, emission at 505–550 nm. Images were processed using the same parameters on LAS AF Lite software (Leica).

### Gene Set Enrichment Analysis (GSEA) and Multidimensional Scaling

We used GSEA v3.0 (http://software.broadinstitute.org/gsea/downloads.jsp) to identify enriched gene sets associated with *KIT* or *NTRK1* gene expression in NB and AML patients. *KIT* and *NTRK1* expression was used as phenotype label and GO biological processes gene sets were analyzed. The Pearson correlation was used for the gene ranking metric, and the number of permutations was set at 100. Enrichment results satisfying a nominal *p* < 0.05 with a false discovery rate (FDR) < 0.25 were considered statistically significant. For the multidimensional scaling of enriched GO gene sets we used a web interface: REVIGO (42). The Resnik score for depth of GO gene annotation was used for multidimensional scaling and the obtained results were then plotted in Cytoscape software (91).

### Identification of Differentially Expressed Genes (DEGs), Survival, and GeneMANIA Analysis

Multiple *t*-tests for genes in NB and AML datasets were performed in GraphPad Prism 8 software. For each data set we divided patients into two groups based on *KIT* or *NTRK1* gene expression. Groups with high *KIT* or *NTRK1* expression for each data set were selected starting from 10 with 5% increments until a considerable number of DEGs were identified. Groups of the NB data set (Versteeg *n* = 88) were 30% (*KIT*-high) and 25% (*NTRK1*-high). Groups of the AML data set (denBoer *n* = 237) were 15% for both *KIT*-high and *NTRK1*-high. Genes with a false discovery rate (FDR *q*-value) < 0.01% were considered as differentially expressed. The Kaplan Meier scan from R2: Genomics analysis and visualization platform was used for overall survival analysis. Differences in overall survival probability with *p* < 0.05 after Bonferroni correction were considered significant. Patients were separated into high and low groups based on gene expression, groups with lowest Bonferroni *p*-value were presented in results. To predict potential interactions between identified genes, we employed the GeneMANIA (http://www.genemania.org/), a Cytoscape (91) application tool used for the functional prediction of gene and protein interactions (92).

### Statistical Analysis

All the data are expressed as mean ± SD from at least three individual experiments. The statistical significance of differences observed in cell count experiments was determined by the Mann-Whitney non-parametric test. The statistical significance of real-time PCR experiments was determined by an unpaired two-sided Student *t*-test. A *p* < 0.05 marks significance. All statistical calculations were performed in GraphPad Prism 8 software.

## Data Availability Statement

The datasets generated for this study are available on request to the corresponding author.

## Author Contributions

EV and TL designed and performed the research, analyzed the data, and wrote the original manuscript. ICC was performed by VIP and OL. GSEA, multidimensional scaling, and statistical analysis was performed by TL. EV developed experimental methodology and supervised the resources. PS and VSP supervised the study and corrected the manuscript.

### Conflict of Interest

The authors declare that the research was conducted in the absence of any commercial or financial relationships that could be construed as a potential conflict of interest.

## Supplementary Material

Supplementary Table 1 Coordinates for all data sets used for 2D-plot of KIT vs NTRK1 expression.

Supplementary Table 2 List of enriched Gene Ontology gene sets identified by GSEA in t(8;21)/inv16 AML, other AML karyotypes and NB with high KIT expression. SIZE- total number of genes in gene set; ES- enrichment score; NES- normalized enrichment score.

Supplementary Table 3 List of enriched Gene Ontology gene sets identified by GSEA in t(8;21)/inv16 AML and NB with high NTRK1 expression. SIZE- total number of gene in gene set; ES- enrichment score; NES- normalized enrichment score.

Supplementary Table 4 List of differentially expressed genes in AML groups with KIT high/low and NTRK1 high/low expression.

Supplementary Table 5 List of differentially expressed genes in NB groups with KIT high/low and NTRK1 high/low expression.

Supplementary Table 6 List of primers used for real-time PCR.

## References

[1] Maris JM Hogarty MD Bagatell R Cohn SL. Neuroblastoma. Lancet. (2007) 369:2106–20. 10.1016/S0140-6736(07)60983-0 17586306

[2] de Rooij JD Zwaan CM van den Heuvel-Eibrink M. Pediatric AML: from biology to clinical management. J Clin Med. (2015) 4:127–49. 10.3390/jcm4010127 26237023 PMC4470244

[3] Vagapova ER Spirin PV Lebedev TD Prassolov VS. The role of TAL1 in hematopoiesis and leukemogenesis. Acta Naturae. (2018) 10:15–23. 10.32607/20758251-2018-10-1-15-23 29713515 PMC5916730

[4] Wang YY Zhou GB Yin T Chen B Shi JY Liang WX. AML1-ETO and C-KIT mutation/overexpression in t(8;21) leukemia: implication in stepwise leukemogenesis and response to Gleevec. Proc Natl Acad Sci USA. (2005) 102:1104–9. 10.1073/pnas.0408831102 15650049 PMC545849

[5] Ayatollahi H Shajiei A Sadeghian MH Sheikhi M Yazdandoust E Ghazanfarpour M. Prognostic importance of C-KIT mutations in core binding factor acute myeloid leukemia: a systematic review. Hematol Oncol Stem Cell Ther. (2017) 10:1–7. 10.1016/j.hemonc.2016.08.005 27613372

[6] Voytyuk O Lennartsson J Mogi A Caruana G Courtneidge S Ashman LK. Src family kinases are involved in the differential signaling from two splice forms of c-Kit. J Biol Chem. (2003) 278:9159–66. 10.1074/jbc.M211726200 12511554

[7] Young SM Cambareri AC Odell A Geary SM Ashman LK. Early myeloid cells expressing c-KIT isoforms differ in signal transduction, survival and chemotactic responses to stem cell factor. Cell Signal. (2007) 19:2572–81. 10.1016/j.cellsig.2007.08.004 17855052

[8] Cohen PS Chan JP Lipkunskaya M Biedler JL Seeger RC. Expression of stem cell factor and c-kit in human neuroblastoma. The Children's Cancer Group. Blood. (1994) 84:3465–72. 7524740

[9] Vitali R Cesi V Nicotra MR McDowell HP Donfrancesco A Mannarino O. c-Kit is preferentially expressed in MYCN-amplified neuroblastoma and its effect on cell proliferation is inhibited *in vitro* by STI-571. Int J Cancer. (2003) 106:147–52. 10.1002/ijc.11187 12800187

[10] Krams M Parwaresch R Sipos B Heidorn K Harms D Rudolph P. Expression of the c-kit receptor characterizes a subset of neuroblastomas with favorable prognosis. Oncogene. (2004) 23:588–95. 10.1038/sj.onc.1207145 14724587

[11] Uccini S Mannarino O McDowell HP Pauser U Vitali R Natali PG. Clinical and molecular evidence for c-kit receptor as a therapeutic target in neuroblastic tumors. Clin Cancer Res. (2005) 11:380–9. 15671569

[12] Shimada A Hirato J Kuroiwa M Kikuchi A Hanada R Wakai K. Expression of KIT and PDGFR is associated with a good prognosis in neuroblastoma. Pediatr Blood Cancer. (2008) 50:213–7. 10.1002/pbc.21288 17941064

[13] Lau ST Hansford LM Chan WK Chan GC Wan TS Wong KK. Prokineticin signaling is required for the maintenance of a de novo population of c-KIT(+) cells to sustain neuroblastoma progression. Oncogene. (2015) 34:1019–34. 10.1038/onc.2014.24 24632619

[14] Lebedev TD Spirin PV Orlova NN Prokofjeva MM Prassolov VS. [Comparative study of therapy targeted genes expression in neuroblastoma cell lines]. Mol Biol. (2015) 49:1048–51. 10.1134/S0026893315050222 26710789

[15] Orr-Urtreger A Avivi A Zimmer Y Givol D Yarden Y Lonai P. Developmental expression of c-kit, a proto-oncogene encoded by the W locus. Development. (1990) 109:911–23. 1699718 10.1242/dev.109.4.911

[16] Reid K Nishikawa S Bartlett PF Murphy M. Steel factor directs melanocyte development *in vitro* through selective regulation of the number of c-kit+ progenitors. Dev Biol. (1995) 169:568–79. 10.1006/dbio.1995.1170 7540155

[17] Luo R Gao J Wehrle-Haller B Henion PD. Molecular identification of distinct neurogenic and melanogenic neural crest sublineages. Development. (2003) 130:321–30. 10.1242/dev.00213 12466199

[18] Wilson YM Richards KL Ford-Perriss ML Panthier JJ Murphy M. Neural crest cell lineage segregation in the mouse neural tube. Development. (2004) 131:6153–62. 10.1242/dev.01533 15548576

[19] Motohashi T Aoki H Chiba K Yoshimura N Kunisada T. Multipotent cell fate of neural crest-like cells derived from embryonic stem cells. Stem Cells. (2007) 25:402–10. 10.1634/stemcells.2006-0323 17038669

[20] Motohashi T Yamanaka K Chiba K Miyajima K Aoki H Hirobe T. Neural crest cells retain their capability for multipotential differentiation even after lineage-restricted stages. Dev Dyn. (2011) 240:1681–93. 10.1002/dvdy.22658 21594952

[21] Hatzistergos KE Takeuchi LM Saur D Seidler B Dymecki SM Mai JJ. cKit+ cardiac progenitors of neural crest origin. Proc Natl Acad Sci USA. (2015) 112:13051–6. 10.1073/pnas.1517201112 26438843 PMC4620867

[22] Lebedev TD Spirin PV Suntsova MV Ivanova AV Buzdin AA Prokofjeva MM. [Receptor tyrosine kinase KIT may regulate expression of genes involved in spontaneous regression of neuroblastoma]. Mol Biol. (2015) 49:1052–5. 10.1134/S0026893315060151 26710790

[23] Rossler J Zambrzycka I Lagodny J Kontny U Niemeyer CM. Effect of STI-571 (imatinib mesylate) in combination with retinoic acid and gamma-irradiation on viability of neuroblastoma cells. Biochem Biophys Res Commun. (2006) 342:1405–12. 10.1016/j.bbrc.2006.02.059 16524560

[24] Timeus F Crescenzio N Fandi A Doria A Foglia L Cordero di Montezemolo L. *In vitro* antiproliferative and antimigratory activity of dasatinib in neuroblastoma and Ewing sarcoma cell lines. Oncol Rep. (2008) 19:353–9. 10.3892/or.19.2.353 18202781

[25] Nakagawara A Arima M Azar CG Scavarda NJ Brodeur GM. Inverse relationship between trk expression and N-myc amplification in human neuroblastomas. Cancer Res. (1992) 52:1364–8. 1737399

[26] Nakagawara A Arima-Nakagawara M Scavarda NJ Azar CG Cantor AB Brodeur GM. Association between high levels of expression of the TRK gene and favorable outcome in human neuroblastoma. N Engl J Med. (1993) 328:847–54. 10.1056/NEJM199303253281205 8441429

[27] Brodeur GM Nakagawara A Yamashiro DJ Ikegaki N Liu XG Azar CG. Expression of TrkA, TrkB and TrkC in human neuroblastomas. J Neurooncol. (1997) 31:49–55. 10.1023/A:1005729329526 9049830

[28] Kogner P Barbany G Dominici C Castello MA Raschella G Persson H. Coexpression of messenger RNA for TRK protooncogene and low affinity nerve growth factor receptor in neuroblastoma with favorable prognosis. Cancer Res. (1993) 53:2044–50. 8481906

[29] Suzuki T Bogenmann E Shimada H Stram D Seeger RC. Lack of high-affinity nerve growth factor receptors in aggressive neuroblastomas. J Natl Cancer Inst. (1993) 85:377–84. 10.1093/jnci/85.5.377 8433391

[30] Tacconelli A Farina AR Cappabianca L Desantis G Tessitore A Vetuschi A. TrkA alternative splicing: a regulated tumor-promoting switch in human neuroblastoma. Cancer Cell. (2004) 6:347–60. 10.1016/j.ccr.2004.09.011 15488758

[31] Farina AR Cappabianca L Gneo L Ruggeri P Mackay AR. TrkAIII signals endoplasmic reticulum stress to the mitochondria in neuroblastoma cells, resulting in glycolytic metabolic adaptation. Oncotarget. (2018) 9:8368–90. 10.18632/oncotarget.23618 29492201 PMC5823587

[32] Descamps S Pawlowski V Revillion F Hornez L Hebbar M Boilly B. Expression of nerve growth factor receptors and their prognostic value in human breast cancer. Cancer Res. (2001) 61:4337–40. 11389056

[33] Pasini L Re A Tebaldi T Ricci G Boi S Adami V. TrkA is amplified in malignant melanoma patients and induces an anti-proliferative response in cell lines. BMC Cancer. (2015) 15:777. 10.1186/s12885-015-1791-y 26496938 PMC4619539

[34] Antunes LCM Cartell A de Farias CB Bakos RM Roesler R Schwartsmann G. Tropomyosin-related kinase receptor and neurotrophin expression in cutaneous melanoma is associated with a poor prognosis and decreased survival. Oncology. (2019) 97:26–37. 10.1159/000499384 31071716

[35] Brodie C Gelfand EW. Functional nerve growth factor receptors on human B lymphocytes. Interaction with IL-2. J Immunol. (1992) 148:3492–97. 1316918

[36] Torcia M Bracci-Laudiero L Lucibello M Nencioni L Labardi D Rubartelli A. Nerve growth factor is an autocrine survival factor for memory B lymphocytes. Cell. (1996) 85:345–56. 10.1016/S0092-8674(00)81113-7 8616890

[37] Mulloy JC Jankovic V Wunderlich M Delwel R Cammenga J Krejci O. AML1-ETO fusion protein up-regulates TRKA mRNA expression in human CD34+ cells, allowing nerve growth factor-induced expansion. Proc Natl Acad Sci USA. (2005) 102:4016–21. 10.1073/pnas.0404701102 15731354 PMC554792

[38] Tacconelli A Farina AR Cappabianca L Cea G Panella S Chioda A. TrkAIII expression in the thymus. J Neuroimmunol. (2007) 183:151–61. 10.1016/j.jneuroim.2006.12.005 17241672

[39] Farina AR Cappabianca L Ruggeri P Gneo L Pellegrini C Fargnoli MC. The oncogenic neurotrophin receptor tropomyosin-related kinase variant, TrkAIII. J Exp Clin Cancer Res. (2018) 37:119. 10.1186/s13046-018-0786-3 29914559 PMC6006588

[40] Molenaar JJ Koster J Zwijnenburg DA van Sluis P Valentijn LJ van der Ploeg I. Sequencing of neuroblastoma identifies chromothripsis and defects in neuritogenesis genes. Nature. (2012) 483:589–93. 10.1038/nature10910 22367537

[41] Balgobind BV Van den Heuvel-Eibrink MM De Menezes RX Reinhardt D Hollink IH Arentsen-Peters ST. Evaluation of gene expression signatures predictive of cytogenetic and molecular subtypes of pediatric acute myeloid leukemia. Haematologica. (2011) 96:221–30. 10.3324/haematol.2010.029660 20971820 PMC3031689

[42] Supek F Bosnjak M Skunca N Smuc T. REVIGO summarizes and visualizes long lists of gene ontology terms. PLoS ONE. (2011) 6:e21800. 10.1371/journal.pone.0021800 21789182 PMC3138752

[43] Li Z Herold T He C Valk PJ Chen P Jurinovic V. Identification of a 24-gene prognostic signature that improves the European LeukemiaNet risk classification of acute myeloid leukemia: an international collaborative study. J Clin Oncol. (2013) 31:1172–81. 10.1200/JCO.2012.44.3184 23382473 PMC3595425

[44] Spirin PV Lebedev TD Orlova NN Gornostaeva AS Prokofjeva MM Nikitenko NA. Silencing AML1-ETO gene expression leads to simultaneous activation of both pro-apoptotic and proliferation signaling. Leukemia. (2014) 28:2222–8. 10.1038/leu.2014.130 24727677

[45] Heo SK Noh EK Kim JY Jeong YK Jo JC Choi Y. Targeting c-KIT (CD117) by dasatinib and radotinib promotes acute myeloid leukemia cell death. Sci Rep. (2017) 7:15278. 10.1038/s41598-017-15492-5 29127384 PMC5681687

[46] Herbrich SM Kannan S Nolo RM Hornbaker M Chandra J Zweidler-McKay PA. Characterization of TRKA signaling in acute myeloid leukemia. Oncotarget. (2018) 9:30092–105. 10.18632/oncotarget.25723 30046390 PMC6059018

[47] Shields S Conroy E O'Grady T McGoldrick A Connor K Ward MP. BAG3 promotes tumour cell proliferation by regulating EGFR signal transduction pathways in triple negative breast cancer. Oncotarget. (2018) 9:15673–90. 10.18632/oncotarget.24590 29644001 PMC5884656

[48] Alharbi RA Pettengell R Pandha HS Morgan R. The role of HOX genes in normal hematopoiesis and acute leukemia. Leukemia. (2013) 27:1000–8. 10.1038/leu.2012.356 23212154

[49] Torres CM Biran A Burney MJ Patel H Henser-Brownhill T Cohen AS. The linker histone H1.0 generates epigenetic and functional intratumor heterogeneity. Science. (2016) 353:aaf1644. 10.1126/science.aaf1644 27708074 PMC5131846

[50] Damri O Sade Y Toker L Bersudsky Y Belmaker RH Agam G. Molecular effects of lithium are partially mimicked by inositol-monophosphatase (IMPA)1 knockout mice in a brain region-dependent manner. Eur Neuropsychopharmacol. (2015) 25:425–34. 10.1016/j.euroneuro.2014.06.012 25748680

[51] Lopez-Delisle L Pierre-Eugene C Louis-Brennetot C Surdez D Raynal V Baulande S. Activated ALK signals through the ERK-ETV5-RET pathway to drive neuroblastoma oncogenesis. Oncogene. (2018) 37:1417–29. 10.1038/s41388-017-0039-5 29321660 PMC6168456

[52] Van den Eynden J Umapathy G Ashouri A Cervantes-Madrid D Szydzik J Ruuth K. Phosphoproteome and gene expression profiling of ALK inhibition in neuroblastoma cell lines reveals conserved oncogenic pathways. Sci Signal. (2018) 11:eaar5680. 10.1126/scisignal.aar5680 30459281

[53] Yuyama K Yamamoto H Nakamura K Kato T Sora I Yamamoto T. Resistance of PC12 cells against nitric oxide (NO)-induced toxicity in long-term culture: implication of neuronal NO synthase expression. Neurosci Lett. (2001) 309:169–72. 10.1016/S0304-3940(01)02078-X 11514068

[54] Ketterer K Kong B Frank D Giese NA Bauer A Hoheisel J. Neuromedin U is overexpressed in pancreatic cancer and increases invasiveness via the hepatocyte growth factor c-Met pathway. Cancer Lett. (2009) 277:72–81. 10.1016/j.canlet.2008.11.028 19118941

[55] Lin TY Wu FJ Chang CL Li Z Luo CW. NMU signaling promotes endometrial cancer cell progression by modulating adhesion signaling. Oncotarget. (2016) 7:10228–42. 10.18632/oncotarget.7169 26849234 PMC4891116

[56] Garczyk S Klotz N Szczepanski S Denecke B Antonopoulos W von Stillfried S. Oncogenic features of neuromedin U in breast cancer are associated with NMUR2 expression involving crosstalk with members of the WNT signaling pathway. Oncotarget. (2017) 8:36246–65. 10.18632/oncotarget.16121 28423716 PMC5482652

[57] Hirschmann-Jax C Foster AE Wulf GG Nuchtern JG Jax TW Gobel U. A distinct “side population” of cells with high drug efflux capacity in human tumor cells. Proc Natl Acad Sci USA. (2004) 101:14228–33. 10.1073/pnas.0400067101 15381773 PMC521140

[58] Walton JD Kattan DR Thomas SK Spengler BA Guo HF Biedler JL. Characteristics of stem cells from human neuroblastoma cell lines and in tumors. Neoplasia. (2004) 6:838–45. 10.1593/neo.04310 15720811 PMC1531688

[59] Acosta S Lavarino C Paris R Garcia I de Torres C Rodriguez E. Comprehensive characterization of neuroblastoma cell line subtypes reveals bilineage potential similar to neural crest stem cells. BMC Dev Biol. (2009) 9:12. 10.1186/1471-213X-9-12 19216736 PMC2647534

[60] Ross RA Walton JD Han D Guo HF Cheung NK. A distinct gene expression signature characterizes human neuroblastoma cancer stem cells. Stem Cell Res. (2015) 15:419–26. 10.1016/j.scr.2015.08.008 26342562 PMC4601571

[61] Farina AR Tacconelli A Cappabianca L Cea G Panella S Chioda A. The alternative TrkAIII splice variant targets the centrosome and promotes genetic instability. Mol Cell Biol. (2009) 29:4812–30. 10.1128/MCB.00352-09 19564412 PMC2725721

[62] Farina AR Cappabianca L Ruggeri P Gneo L Maccarone R Mackay AR. Retrograde TrkAIII transport from ERGIC to ER: a re-localisation mechanism for oncogenic activity. Oncotarget. (2015) 6:35636–51. 10.18632/oncotarget.5802 26415233 PMC4742131

[63] Pugh TJ Morozova O Attiyeh EF Asgharzadeh S Wei JS Auclair D. The genetic landscape of high-risk neuroblastoma. Nat Genet. (2013) 45:279–84. 10.1038/ng.2529 23334666 PMC3682833

[64] Li X Li C Jin J Wang J Huang J Ma Z. High PARP-1 expression predicts poor survival in acute myeloid leukemia and PARP-1 inhibitor and SAHA-bendamustine hybrid inhibitor combination treatment synergistically enhances anti-tumor effects. EBioMed. (2018) 38:47–56. 10.1016/j.ebiom.2018.11.025 30472087 PMC6306376

[65] Petrov I Suntsova M Ilnitskaya E Roumiantsev S Sorokin M Garazha A. Gene expression and molecular pathway activation signatures of MYCN-amplified neuroblastomas. Oncotarget. (2017) 8:83768–80. 10.18632/oncotarget.19662 29137381 PMC5663553

[66] Herold S Kalb J Buchel G Ade CP Baluapuri A Xu J. Recruitment of BRCA1 limits MYCN-driven accumulation of stalled RNA polymerase. Nature. (2019) 567:545–9. 10.1038/s41586-019-1030-9 30894746 PMC7611299

[67] Auffray I Chevalier S Froger J Izac B Vainchenker W Gascan H. Nerve growth factor is involved in the supportive effect by bone marrow–derived stromal cells of the factor-dependent human cell line UT-7. Blood. (1996) 88:1608–18. 8781416

[68] Garcia R Aguiar J Alberti E de la Cuetara K Pavon N. Bone marrow stromal cells produce nerve growth factor and glial cell line-derived neurotrophic factors. Biochem Biophys Res Commun. (2004) 316:753–4. 10.1016/j.bbrc.2004.02.111 15033464

[69] Xie P Cheung WM Ip FC Ip NY Leung MF. Induction of TrkA receptor by retinoic acid in leukaemia cell lines. Neuroreport. (1997) 8:1067–70. 10.1097/00001756-199703240-00001 9175086

[70] Xie P Chan FS Ip NY Leung MF. Induction of TrkA expression by differentiation inducers in human myeloid leukemia KG-1 cells. Leuk Lymphoma. (2000) 36:595–601. 10.3109/10428190009148408 10784405

[71] Tsuda T Wong D Dolovich J Bienenstock J Marshall J Denburg JA. Synergistic effects of nerve growth factor and granulocyte-macrophage colony-stimulating factor on human basophilic cell differentiation. Blood. (1991) 77:971–9. 1995103

[72] Xie P Chan FS Ip NY Leung M. Nerve growth factor potentiated the sodium butyrate- and PMA-induced megakaryocytic differentiation of K562 leukemia cells. Leuk Res. (2000) 24:751–9. 10.1016/S0145-2126(00)00044-8 10978779

[73] Koch A Scherr M Breyer B Mancini A Kardinal C Battmer K. Inhibition of Abl tyrosine kinase enhances nerve growth factor-mediated signaling in Bcr-Abl transformed cells via the alteration of signaling complex and the receptor turnover. Oncogene. (2008) 27:4678–89. 10.1038/onc.2008.107 18427551

[74] Li Z Beutel G Rhein M Meyer J Koenecke C Neumann T. High-affinity neurotrophin receptors and ligands promote leukemogenesis. Blood. (2009) 113:2028–37. 10.1182/blood-2008-05-155200 19059881 PMC2651014

[75] Pardanani A. JAK2 inhibitor therapy in myeloproliferative disorders: rationale, preclinical studies and ongoing clinical trials. Leukemia. (2008) 22:23–30. 10.1038/sj.leu.2404948 17882282

[76] Shimada H Nakagawa A Peters J Wang H Wakamatsu PK Lukens JN. TrkA expression in peripheral neuroblastic tumors: prognostic significance and biological relevance. Cancer. (2004) 101:1873–81. 10.1002/cncr.20557 15386308

[77] Kim CJ Matsuo T Lee KH Thiele CJ Up-regulation of insulin-like growth factor-II expression is a feature of TrkA but not TrkB activation in SH-SY5Y neuroblastoma cells. Am J Pathol. (1999) 155:1661–70. 10.1016/S0002-9440(10)65481-8 10550322 PMC1866969

[78] Light JE Koyama H Minturn JE Ho R Simpson AM Iyer R. Clinical significance of NTRK family gene expression in neuroblastomas. Pediatr Blood Cancer. (2012) 59:226–32. 10.1002/pbc.23343 21990266 PMC3258457

[79] Li J Feng D Gao C Zhang Y Xu J Wu M. Isoforms S and L of MRPL33 from alternative splicing have isoformspecific roles in the chemoresponse to epirubicin in gastric cancer cells via the PI3K/AKT signaling pathway. Int J Oncol. (2019) 54:1591–600. 10.3892/ijo.2019.4728 30816492 PMC6438423

[80] Behrens K Triviai I Schwieger M Tekin N Alawi M Spohn M. Runx1 downregulates stem cell and megakaryocytic transcription programs that support niche interactions. Blood. (2016) 127:3369–81. 10.1182/blood-2015-09-668129 27076172

[81] Chen Y Soong J Mohanty S Xu L Scott G. The neural guidance receptor Plexin C1 delays melanoma progression. Oncogene. (2013) 32:4941–9. 10.1038/onc.2012.511 23160370 PMC3758461

[82] Dwyer DF Barrett NA Austen KF. Expression profiling of constitutive mast cells reveals a unique identity within the immune system. Nat Immunol. (2016) 17:878–87. 10.1038/ni.3445 27135604 PMC5045264

[83] Bhatlekar S Fields JZ Boman BM. Role of HOX genes in stem cell differentiation and cancer. Stem Cells Int. (2018) 2018:3569493. 10.1155/2018/3569493 30154863 PMC6081605

[84] Wurm M Kowalski J Heckl D Zhang XB Nelson V Beard BC. Ectopic expression of HOXC6 blocks myeloid differentiation and predisposes to malignant transformation. Exp Hematol. (2014) 42:114–25.e114. 10.1016/j.exphem.2013.10.004 24513167 PMC4062101

[85] You S Gao L. Identification of NMU as a potential gene conferring alectinib resistance in non-small cell lung cancer based on bioinformatics analyses. Gene. (2018) 678:137–42. 10.1016/j.gene.2018.08.032 30096454

[86] Shetzline SE Rallapalli R Dowd KJ Zou S Nakata Y Swider CR. Neuromedin U: a Myb-regulated autocrine growth factor for human myeloid leukemias. Blood. (2004) 104:1833–40. 10.1182/blood-2003-10-3577 15187020

[87] Ciani E Severi S Contestabile A Bartesaghi R. Nitric oxide negatively regulates proliferation and promotes neuronal differentiation through N-Myc downregulation. J. Cell Sci. (2004) 117(Pt 20):4727–37. 10.1242/jcs.01348 15331636

[88] Vaz GC Sharma NM Zheng H Zimmerman MC Santos RS Frezard F. Liposome-entrapped GABA modulates the expression of nNOS in NG108-15 cells. J Neurosci Methods. (2016) 273:55–63. 10.1016/j.jneumeth.2016.08.004 27523033 PMC5075514

[89] Roberts SS Mori M Pattee P Lapidus J Mathews R O'Malley JP. GABAergic system gene expression predicts clinical outcome in patients with neuroblastoma. J Clin Oncol. (2004) 22:4127–34. 10.1200/JCO.2004.02.032 15483022

[90] Hackett CS Quigley DA Wong RA Chen J Cheng C Song YK. Expression quantitative trait loci and receptor pharmacology implicate Arg1 and the GABA-A receptor as therapeutic targets in neuroblastoma. Cell Rep. (2014) 9:1034–46. 10.1016/j.celrep.2014.09.046 25437558 PMC4251494

[91] Shannon P Markiel A Ozier O Baliga NS Wang JT Ramage D. Cytoscape: a software environment for integrated models of biomolecular interaction networks. Genome Res. (2003) 13:2498–504. 10.1101/gr.1239303 14597658 PMC403769

[92] Warde-Farley D Donaldson SL Comes O Zuberi K Badrawi R Chao P. The GeneMANIA prediction server: biological network integration for gene prioritization and predicting gene function. Nucleic Acids Res. (2010) 38:W214–20. 10.1093/nar/gkq537 20576703 PMC2896186

